# Imaging and milling resolution of light ion beams from helium ion microscopy and FIBs driven by liquid metal alloy ion sources

**DOI:** 10.3762/bjnano.11.156

**Published:** 2020-11-18

**Authors:** Nico Klingner, Gregor Hlawacek, Paul Mazarov, Wolfgang Pilz, Fabian Meyer, Lothar Bischoff

**Affiliations:** 1 Helmholtz-Zentrum Dresden-Rossendorf, Institute of Ion Beam Physics and Materials Research, Bautzner Landstrasse 400, 01328 Dresden, Germany; 2 Raith GmbH, Konrad-Adenauer-Allee 8, 44263 Dortmund, Germany

**Keywords:** focused ion beam, gas field ion source, liquid metal alloy ion source, resolution

## Abstract

While the application of focused ion beam (FIB) techniques has become a well-established technique in research and development for patterning and prototyping on the nanometer scale, there is still a large underused potential with respect to the usage of ion species other than gallium. Light ions in the range of *m* = 1–28 u (hydrogen to silicon) are of increasing interest due to the available high beam resolution in the nanometer range and their special chemical and physical behavior in the substrate. In this work, helium and neon ion beams from a helium ion microscope are compared with ion beams such as lithium, beryllium, boron, and silicon, obtained from a mass-separated FIB using a liquid metal alloy ion source (LMAIS) with respect to the imaging and milling resolution, as well as the current stability. Simulations were carried out to investigate whether the experimentally smallest ion-milled trenches are limited by the size of the collision cascade. While He^+^ offers, experimentally and in simulations, the smallest minimum trench width, light ion species such as Li^+^ or Be^+^ from a LMAIS offer higher milling rates and ion currents while outperforming the milling resolution of Ne^+^ from a gas field ion source. The comparison allows one to select the best possible ion species for the specific demands in terms of resolution, beam current, and volume to be drilled.

## Introduction

In modern nanotechnology, focused ion beam (FIB) techniques are well-established for nanoscale structuring, local surface modification, doping, prototyping, as well as for ion beam analysis. One of the main components of such a FIB system is the ion source providing the needed ion species [1]. Currently, the majority of such instruments use a gallium liquid metal ion source (Ga-LMIS), but the demand in research as well as in the industry for other ion species is increasing permanently. Today, nearly half of the elements of the periodic table are demonstrated to be usable in FIB applications [2]. In particular, light elements in the mass range of *m* = 1–28 u (hydrogen to silicon) and energies between a few and 80 kiloelectronvolts are of special interest. The combination of this energy range with the particular mass range allows one to reach single-digit nanometer and even sub-nanometer resolution. This mass range is of interest due to the interaction of the ions with the near-surface region and, among other use cases, the application of these ions for indirect or resist-aided lithography [3]. The introduction of the helium ion microscope (HIM) [4], working with a gas field ion source (GFIS), about ten years ago solved a lot of problems in this field. However, besides the excellent resolution of the beam, there are also some disadvantages such as small available currents in the low-picoampere range, low number of sputtered ions per incident ion (sputter yield) during structuring, or the formation of helium bubbles in the substrate when using high fluences [5]. In addition to many imaging applications, HIM has been used to create and study new device concepts, including the fabrication of nanometer-sized ferromagnets [6], the controlled tuning of memristive properties of 2D materials [7], the fabrication of graphene nanomeshes [8], the formation of single Si nanocrystals embedded in SiO_2_ for single-electron transistors [9], the spatially resolved engineering of the thermal conductivity in individual Si nanowires [10], as well as the creation of nano-Josephson superconducting tunnel junctions in high-temperature superconductors [11].

Although HIM is highly suitable for imaging and nanometer-scale patterning, there is a need of focused ion beams other than helium or neon with comparable properties. Alternative developments were made using laser-cooled magneto-optical trap ion sources (MOTISs) [12] or classical FIBs equipped with mass separation and liquid metal alloy ion sources working with suitable alloys containing light elements [2].

In the past, mostly heavier ions have been used in liquid metal alloy ion sources (LMAISs). A number of applications have been shown, including using mass-separated FIBs from a Co_36_Nd_64_ LMAIS to implant Co into Si at elevated temperatures, leading to metallic CoSi_2_ nanostructures down to 20 nm [13]. Ge nanowires could be grown by molecular beam epitaxy, via a vapor–liquid–solid process, on a Si substrate after formation of a regular seed array using a mass-separated FIB for Au implantation from a Au_82_Si_18_ LMAIS [14].

**Figure 1:**
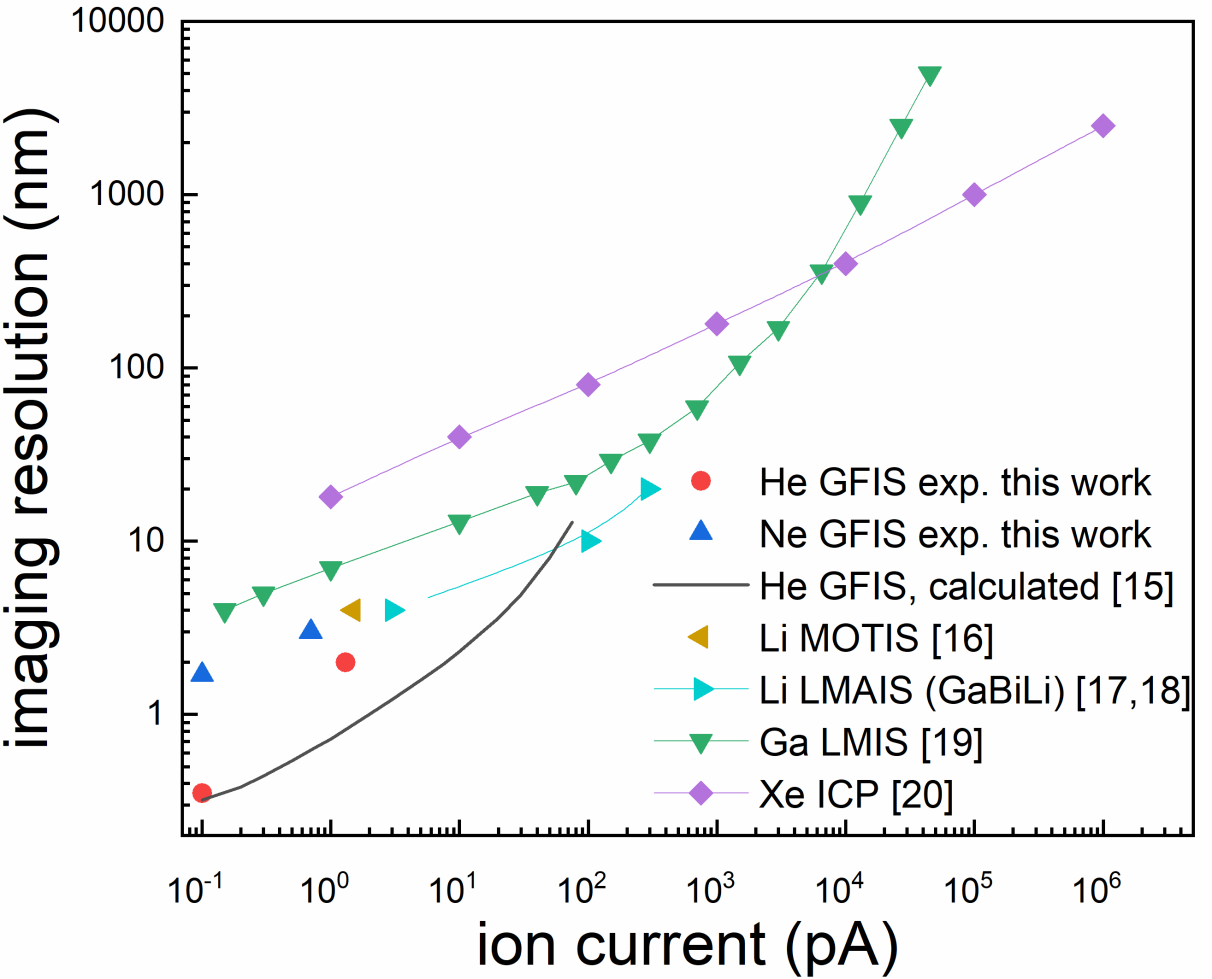
Comparison of the spot size (imaging resolution) as a function of the ion beam current for different relevant FIB systems determined theoretically [15] (r50) and from experiments using a 20% to 80% rise of intensity for He, Li [16–18], Ne, and Ga [19]. The beam size criterion for Xe [20] is unknown.

In this work, the performance of light ion beams from LMAISs will be compared with that of helium and neon ions delivered by the GFIS of a HIM. In Figure 1, a comparison of the performance with respect to the achievable spot size as a function of the beam current for light-ion FIBs and some commercially available FIBs with heavier primary ions is shown. The best values from the literature are evaluated to compare the beam profiles of different ion and source types. Deterioration of the spot size due to vibrations or thermal drift are not explicitly considered in this work, since they are not a fundamental limitation but rather depend on the tool and the setup.

## Experimental

Depending on the application, a FIB column can be operated in different modes. High currents can be obtained in a crossover mode (large-diameter apertures) delivering a weaker resolution [2]. Better resolution requires lower currents (in crossover mode) or a parallel or divergent beam to avoid beam broadening caused by Coulomb interaction. The beam spot size *d* can be described in the following form [2]:

[1] $$d=\sqrt{{\left(Md_{\text{q}}\right)}^{2}+d_{\text{S}}^{2}+d_{\text{C}}^{2}}.$$

Here, *d*_q_ is the virtual source size, which is of utmost importance for the achievable resolution of the FIB. For a LMAIS, it depends on the ion mass and typically has values in the range of 20 to 50 nm [21]. For a GFIS used in a HIM, this value is, under optimal conditions, about 100 times smaller, comparable to the size of a single atom. *M* denotes the magnification of the column [22]. The quantities *d*_S_ and *d*_C_ are defined as the spherical and axial chromatic aberrations, respectively, given as:

[2] $$d_{\text{S}}=\frac{1}{2}C_{\text{S}}\alpha^{3},\;\;\;d_{\text{C}}=C_{\text{C}}\frac{\Delta E}{E}\alpha .$$

Here, *C*_S_ and *C*_C_ are the spherical and chromatic aberration coefficients of the ion optical column, respectively [22]. The quantity α is the acceptance half-angle on the sample and can be determined by α^2^ = I*(π*M*^2^(d*I*/dΩ))^−1^, where *I* is the ion current and d*I*/dΩ is the angular intensity [22]. The energy spread Δ*E* of the ion source is a second key parameter for the final resolution concerning the chromatic aberration. While this value varies for different LMAISs, it is less than 1 eV for a GFIS [23–24]. *E* denotes the primary ion energy. For our special case of interest, that is, light ions and small ion beam currents, the spherical aberration can be neglected and diffraction phenomena, important for electron beams, have no impact [25].

In the following, the discussion will focus on the usage of GFISs and LMAISs in FIBs. GFISs are operated with highly purified helium or neon. No additional mass separation is required in the column. In a LMAIS, in contrast, the source material is a complex alloy delivering several ion species simultaneously in a process of field evaporation. This initially provides a beam containing ions with different masses and charge states, whose fractions depend on the composition of the alloy and the ionization probability. To overcome this problem, an ExB mass filter is introduced in the ion optical column, described in more detail in [2]. In principle, an ExB filter acts as a velocity filter and splits the beam according to the mass-to-charge ratio of its constituents. The mass resolution *m*/Δ*m* of an ExB filter can be written in the following form [26]:

[3] $$\frac{m}{\Delta m}=\frac{E_{x}}{2U_{\text{acc}}}\frac{l}{d}\left(\frac{1}{2}l+D\right).$$

Here, *U*_acc_ is the acceleration voltage of the ion beam and *E_x_* is the electric field strength of the filter perpendicular to the optical axis of the ion column. *l* is the filter length, *D* is the distance between the filter and the separation aperture at the exit of the filter, and *d* is the diameter of this aperture.

**Figure 2:**
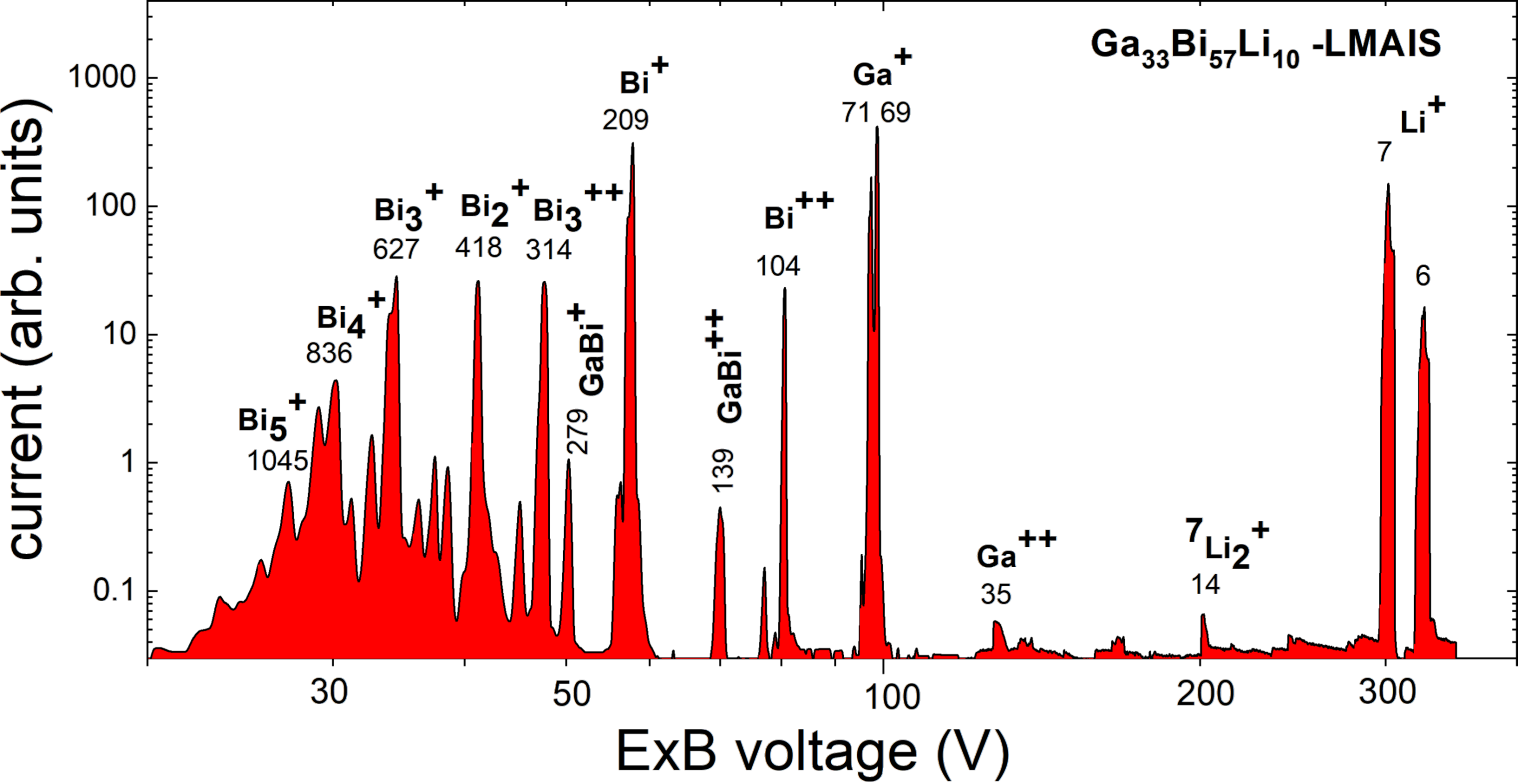
Mass spectrum of a Ga_33_Bi_57_Li_10_ LMAIS at an acceleration potential of 10 kV scanned by the ExB voltage using a constant magnetic field. The numbers below the ion label specify the total ion mass *m* in u.

As an example for a LMAIS mass spectrum, we show results obtained with a Ga_33_Bi_57_Li_10_ source [17] in Figure 2. The used FIB system is equipped with a retarding field analyzer and is described elsewhere [27]. This setup allows one to determine the energy spread of all ion species that can be extracted from the LMAIS. This is important to determine the chromatic aberration, which strongly influences the achievable resolution of the FIB. The current was measured using a secondary electron multiplier and is therefore given in arbitrary units. For the light isotope ^6^Li^+^ at 1 μA emission current a Δ*E* of 3 eV could be determined [17], which is in a good agreement with the Δ*E* ~ *m*^1/3^ dependence for single-charged monomers [2].

In the noble gas irradiation experiments described here, a GFIS in a helium ion microscope ORION NanoFab (Carl Zeiss) [5,28] was used. For irradiation with light metal ions, a LMAIS installed in a FIB/SEM VELION system (Raith) [2,18,29] was used. All milling experiments were performed on a 100 nm thin gold film on glass. For measuring the trench width milled into the gold layer, either the same primary ion beam microscope or a scanning electron microscope have been used to image the sputtered trenches. The investigated sources are listed in Table 1. Details of fabrication and operation principle are given in the corresponding references.

**Table 1:** Light ion species, source type, source temperature for GFIS and LMAIS, source material, emitter tip material, and corresponding references. For the LMAIS, the listed temperatures are the melting points of the eutectic alloy. The actually used temperatures are a little higher.

Light ion species	Source type	*T*_operation_ (K)	Material	Emitter	Ref.

He^+^, Ne^+^	GFIS	80	He, Ne gas	W	[4–5]
Li^+^	LMAIS	495	Ga_35_Bi_60_Li_5_	W	[18]
Be^+^, Be^++^	LMAIS	640	Au_70_Si_15_Be_15_	W	[30]
B^+^, B^++^	LMAIS	920	Co_31_Nd_64_B_5_	Ta, W	[31]
C^+^	LMAIS	933	C_20_Al_10_Ce_70_	Ta	[32]
Si^+^, Si^++^	LMAIS	638	Au_82_Si_18_	W	[33]

## Results and Discussion

Resolution values obtained with different light ion species on a Au film are summarized in Table 2. The sputter yields *Y*_theo_ for normal incidence based on the work of Yamamura et al. [34] are also given in Table 2. They are in a good agreement with the experimentally determined data from literature (volume-loss method), for example, for 30 keV helium in gold *Y*_exp_ = 0.13 and for 35 keV lithium in gold *Y*_exp_ = 0.4 [17]. Single-pixel lines have been milled with different fluence values using small ion currents to get the best possible resolution. The imaging resolution was determined by sweeping the beam over a sharp border in a one-line scan and examining the slope in 80/20 intensity mode. For determining the imaging resolution in HIM, few pixel wide line profiles are made across selected edges in previously recorded images with an appropriate pixel size and are evaluated according to the same 80/20 criterion.

**Table 2:** Irradiation parameters for several available ions, calculated sputter yields *Y*_theo_ [34] of Au, imaging resolution (80/20 criterion), the smallest achieved milling trench width and typical ion beam currents as well as their stability logged over several hours. Literature values are indicated by their reference, data from this work is marked by an asterisk.

Source	Ion	*Y*_theo_ [33] on Au	Imaging resolution	Trench width	Ion beam current stability

GFIS					

	30 keV ^4^He^+^	0.12	(0.38 ± 0.08) nm [5]	5 nm [5]	(0.54 ± 0.01) pA 1.9% over 1 h *
	25 keV ^20^Ne^+^	5.4	2.85 nm [35]	12 nm *	(0.64 ± 0.04) pA 6.3% over 1 h *

LMAIS					

CoNdB	35 keV ^11^B^+^	1.74	(19 ± 2) nm *	no data	(19.2 ± 0.4) pA 2.1% over 2.5h [31]
GaBiLi	35 keV ^7^Li^+^	0.5	(2.9 ± 0.5) nm *	6 nm *	(2.89 ± 0.08) pA 2.8% over 16 h *
	35 keV ^209^Bi^+^	27	11 nm *	50 nm [36]	(228 ± 1.6) pA 0.7% over 10 h *
Ga	35 keV 70Ga+	18	2 nm [37]	8.4 nm [38]	(19 ± 0.2) pA 1.1% over 67 h *
AuGeSi	70 keV ^74^Ge^++^	21	10 nm *	20 nm *	(14 ± 0.5) pA 3.6% over 15 h *
AuSi	70 keV ^197^Au^++^	35	10–15 nm [30]	19 nm [38]	(7 ± 0.2) pA 2.9% over 25 h [30]
	70 keV ^28^Si^++^	8.42	6–10 nm [30]	13 nm [38]	(12 ± 0.4) pA 3.3% over 15 h *
AuSiBe	70 keV 9Be++	0.84	3–4 nm [30]	7 nm [38]	(6.2 ± 0.1) pA 1.6% over 10 h *

**Figure 3:**
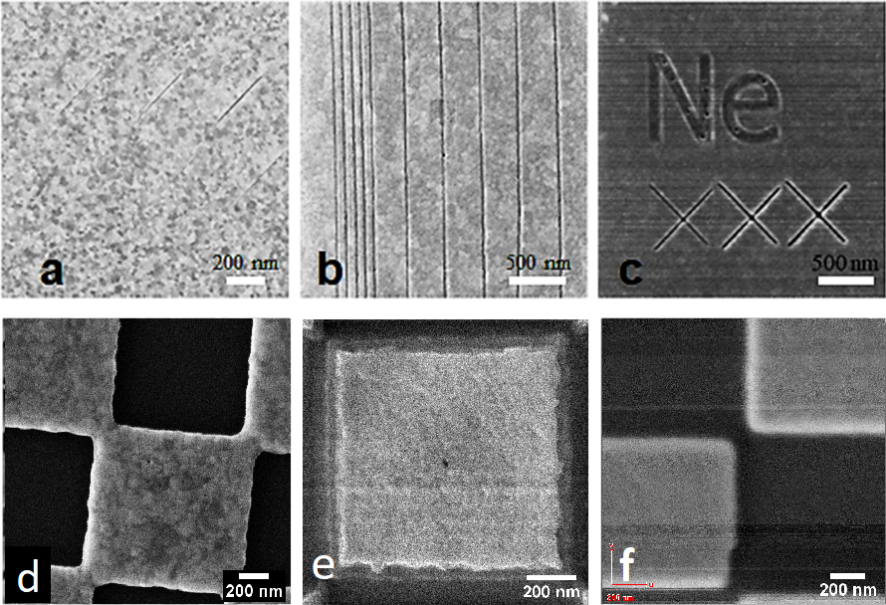
Examples using different ions: a) 30 keV He^+^, field of view (FOV): 1.5 × 1.5 µm^2^, trench width: 4 nm, b) 40 keV Li^+^, FOV: 2.5 × 2.5 µm^2^, trench width: 6 nm, c) 25 keV Ne^+^, FOV: 2.5 × 2.5 µm^2^, trench width: 12 nm, d) 70 keV Be^++^, FOV: 1.9 × 1.9 µm^2^, best imaging res: (4.0 ± 0.5) nm, e) 35 keV Li^+^, FOV: 1.2 × 1.2 µm^2^, best imaging res: (2.9 ± 0.5) nm, f) 35 keV B^+^, FOV: 1.6 × 1.6 µm^2^, best imaging res: (19 ± 2) nm.

Representative images used to obtain the results in Table 2 are shown in Figure 3. They should be exemplary for demonstrating the imaging and milling resolution of the different ion beams. Figure 3 also shows the best imaging resolution for lithium and boron published so far. Examples for the other ion species can be found in the references listed in Table 2.

**Figure 4:**
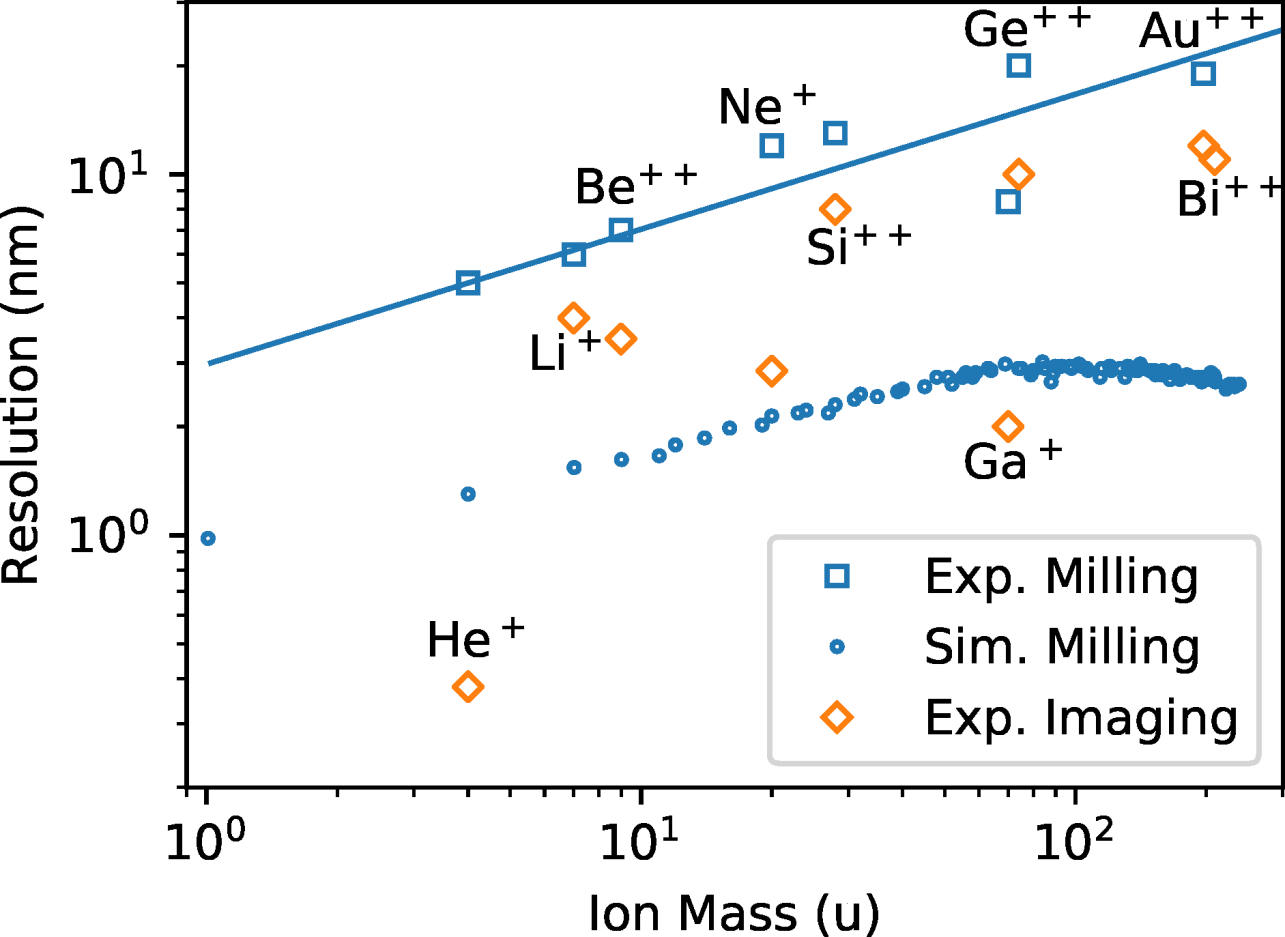
Summary of the imaging resolution (80/20), experimentally achieved trench width, and simulated minimum milling width (FWHM) for FIBs working with different ion species and technologies depending on the ion mass. The line serves as a guide to the eye.

The resolution results for the different ion species listed in Table 2 are plotted in Figure 4. The minimum milling width in dependence of the ion mass, determined from sputtered features (lines, holes), follows a weak linear increase in the double-logarithmic plot. In the case of the imaging resolution the behavior is not so clear. There is a strong variation of the results, due to different beam profiles and ion optical columns, but the imaging resolution is always better than the corresponding milling resolution. The relative difference between the resolution in the two different modes is more pronounced for the GFIS compared to the LMAIS, that is, from 5 nm (milling) to 0.4 nm (imaging) for helium (13 fold) but only from 6 nm (milling) to 4 nm (imaging) for lithium (1.5 fold).

**Figure 5:**
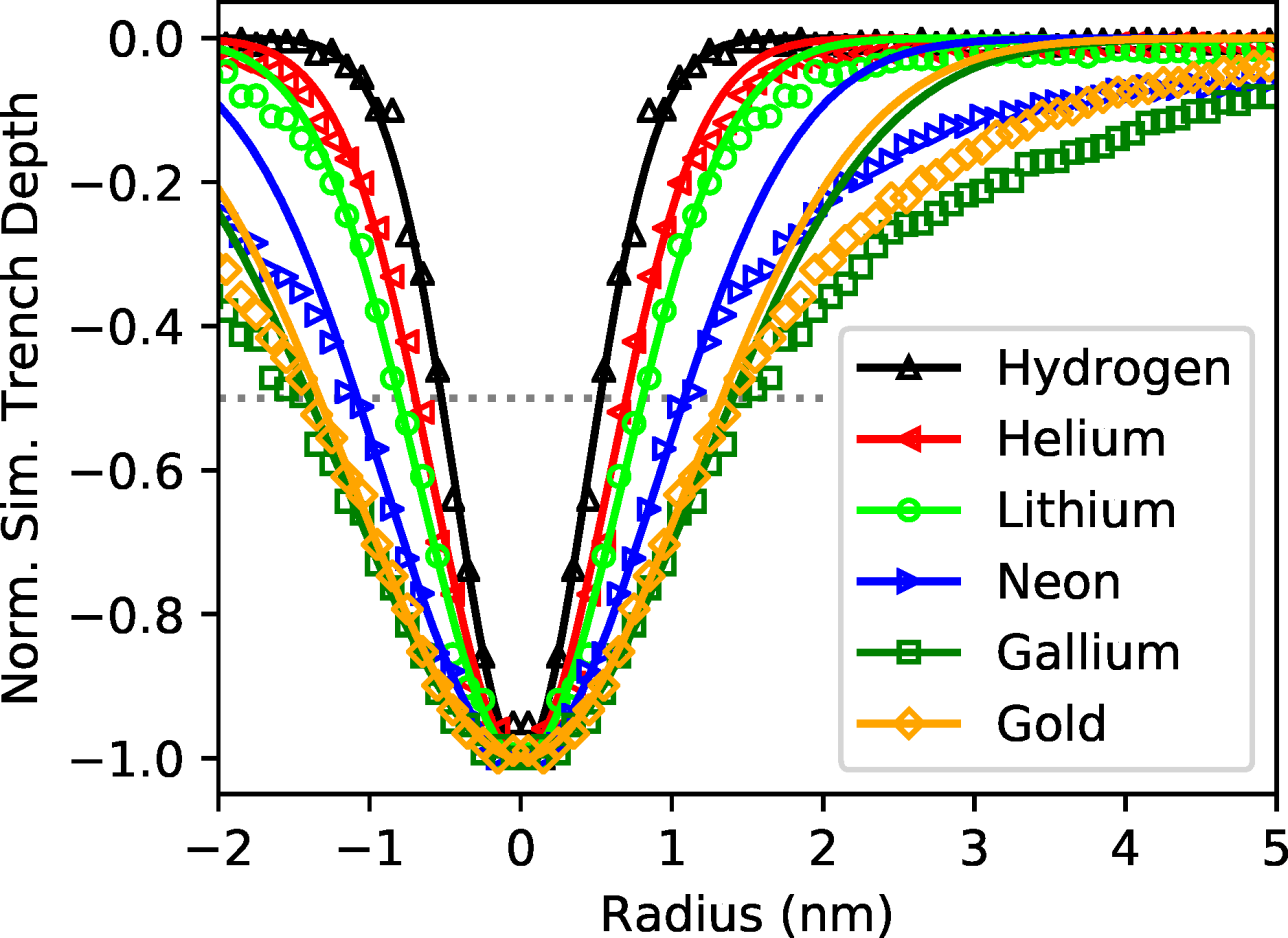
SRIM simulation [39] of the sputter profile from a 30 keV point-like beam in a gold substrate as a function of the ion mass. The dotted line denotes 50% trench depth and corresponds to the minimum milling width of a point-like ion beam.

The simulated minimum milling width of a 30 keV point-like ion beam in Figure 4 has been obtained using SRIM [39]. The “monolayer collision steps/surface sputtering” mode has been used to simulate the size of the collision cascade and the origin of sputtered particles. From 2.5 × 10^5^ ion impacts, for light elements such as hydrogen, down to 5000 ion impacts, for uranium, have been simulated (1 × 10^6^ in total for all elements). The emission position of the sputtered particles (8.5 × 10^6^ in total for all elements) has been evaluated in terms of the distance to the impinging ion. To account for the milling of a line we calculated the projected distance instead of evaluating the radial distance of the emission site from the beam center. The resulting Gaussian-like sputtering profiles have been normalized and analyzed for the full width of the profile at half height or corresponding trench depth (FWHM). The normalized expected trench profiles from a point-like beam in a gold substrate have been plotted for selected ion species in Figure 5. The beam profiles however, are not Gaussian and the edge profiles cannot be described by simple error functions due to the enhanced secondary electron yield on the sample edges. While the imaging resolution was measured using the 80/20 criteria, this is not suitable for trenches. To compare the image resolution with the trench width, for Gaussian profiles, the reader would have to multiply the 20/80 resolution value by a factor of 1.39.

The simulated minimum milling widths are clearly smaller than the experimentally achieved results for all ion species. The main limiting factor for most of them is the spot profile of the ion beam itself. The two exceptions are helium and gallium for which the spot size should be smaller than the simulated minimum milling width. For 30 keV helium in gold, a spot size of 0.38 nm [5] is smaller than the simulated minimum milling of 1.3 nm and much smaller than the smallest achieved trenches of 5 nm [5]. An explanation could be the neglected ion beam stability, mechanical vibrations, drift, or electromagnetic fields during realistic long-term milling processes for light ions due to the small sputter yield (0.12 for 30 keV He in Au [34]). Additionally, large fluences, necessary for deep milling, often cannot be applied when using light ions since the implantation will lead to bubble formation in the target material [40–41]. Gallium in gold has a larger sputter yield of 18 [34] and much higher beam currents are available. Both significantly reduce the milling time and, consequently, the influence of mechanical or electronic drifts. In addition, gas bubble formation will not take place when Ga ions are used.

To discuss the deviation between the simulated minimum milling width and the achieved trench widths in more detail, the experimental sputter beam profiles have been analyzed further. For HIM, the profiles of the helium beam were measured by examining sputtered lines [42] as well as pores [43] in membranes. Gallium is used and optimized for industrial and scientific applications and sputtering beam profiles were measured by TEM [44–45]. The additional influence of the required ExB filter for multi-element or multi-isotope LMAISs is a further factor of uncertainty that, in general, will worsen the achievable spatial resolution.

**Figure 6:**
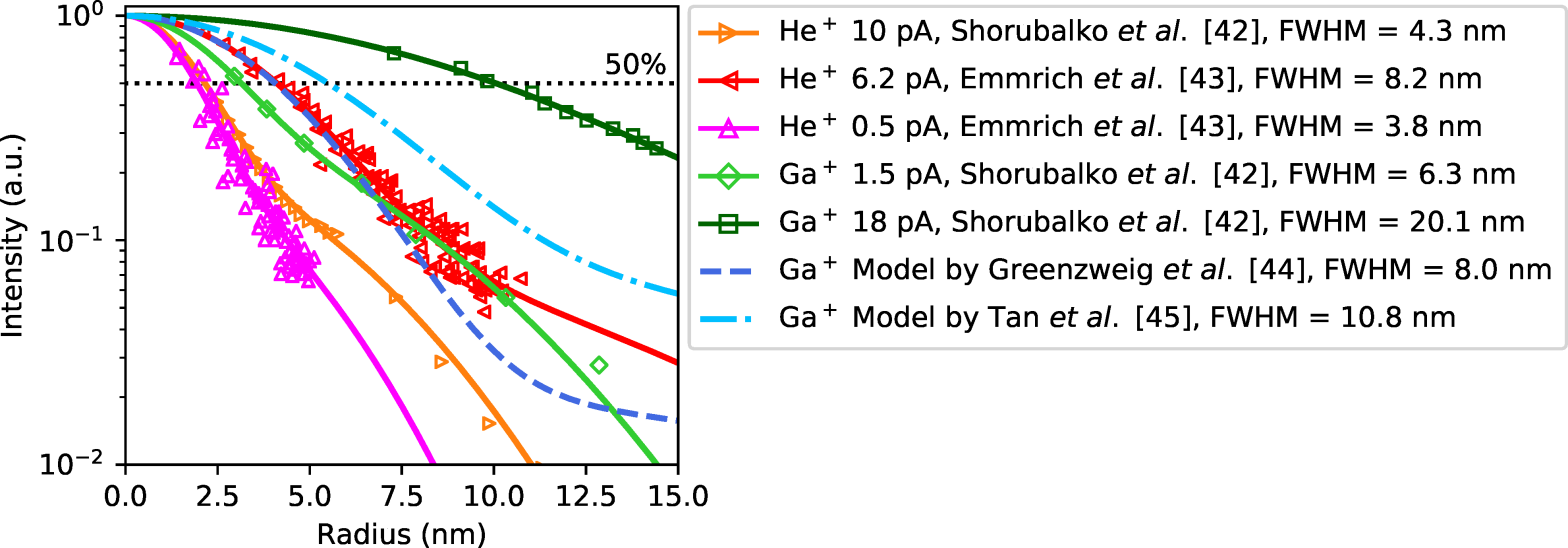
Comparison of the normalized ion beam profiles (normalized half profiles = beam radius) obtained from 1/fluence-dependent milled structures of a helium beam (HIM) on different targets [42–43] and a Ga-LMIS driven FIB [44–45].

Literature data for He and Ga are compiled in Figure 6. Normalized half profiles (ion beam radius) for helium beams averaged for different substrates (taken from [42–43]) and for 40 keV Ga beams on a crystalline Si sample from different studies [44–45] are plotted and fitted by a double Gaussian for comparison in Figure 6. The near-axis resolution of the He beam from a GFIS is smaller than that of the LMIS-driven Ga FIB but the beam tails lead to a comparable behavior along the profile with increasing fluences and, correspondingly, higher milling rates.

The beam tails visible in the sputtering profiles are not visible in the 20/80 imaging resolution tests. This is one contribution leading to larger trenches than expected from the image resolution. Another explanation why the spot size is always smaller than the measured trench width could be the different sample types used for the measurements. While ultimate imaging resolutions are typically measured as an edge contrast on thin freestanding membranes, trenches are typically milled in a metal layer of a few nanometers thickness. Milling membranes removes their support from one side and dangling bonds can lead to morphological changes making milling tests difficult to interpret. In metal layers of a few nanometers thickness, sputter redeposition can take place, which is not taken into account in our static SRIM simulation. As mentioned above the milling of trenches also takes much longer than recording line profiles for capturing SE images, leading to an increased susceptibility for mechanical and electromechanical disturbances.

## Conclusion

In this article, the performance of light ion beams from LMAIS FIBs in terms of imaging resolution and minimum milling width were compared with those of helium and neon beams provided by a GFIS-driven HIM system. According to our simulations and experiments, the imaging and milling resolution of all systems is determined by the ion beam profile and the stability of the ion beam and the instrument itself. While GFIS-driven noble gas beams still deliver the best lateral resolution, LMAISs allow for a wider application spectrum due to the vast number of different ion species and charge states available. Especially for very light ions, such as Li, LMAIS FIBs provide nearly the same milling resolution. Improvement is possible in both cases as GFIS technology can be extended to other gases [46–48]. Resolution improvements should be obtainable for both technologies using better optics and optimized platforms.
